# Dental Erosion in Obese Patients before and after Bariatric Surgery: A Cross-Sectional Study

**DOI:** 10.3390/jcm10214902

**Published:** 2021-10-24

**Authors:** Cui Yang, Frederik Johannes Hammer, Christoph Reissfelder, Mirko Otto, Georgi Vassilev

**Affiliations:** 1 Department of Surgery, Medical Faculty Mannheim, University of Heidelberg, Theodor-Kutzer-Ufer 1-3, 68167 Mannheim, Germany; christoph.reissfelder@umm.de (F.J.H.); mirko.otto@umm.de (C.R.); georgi.vassilev@umm.de (M.O.); frederik.hammer@web.de (G.V.); 2 Faculty of Dentistry, University of Heidelberg, 68167 Mannheim, Germany

**Keywords:** obesity, follow-up, substitution, micronutrient deficiency, dental health, RYGB, VSG, sleeve gastrectomy

## Abstract

Obese patients are at risk of dental erosion due to micronutrient deficiency, consumption of soft drinks, gastric reflux disease and vomiting. The present study evaluates the presence of dental erosion in obese patients before and after bariatric surgery using the BEWE (basic erosive wear examination) scoring system. A total of 62 patients with severe obesity were included in the analysis, 31 in the control group (without bariatric surgery) and 31 in the surgery group (after bariatric surgery). BEWE scores did not vary between groups. Vitamin D deficiency was detected in 19 patients in the control group and three in the surgery group (*p* < 0.001). The serum calcium and vitamin D values were significantly higher in the surgery group (*p* = 0.003, *p* < 0.001 consecutively). All patients after bariatric surgery showed compliance with supplements, including vitamin D and calcium daily. Patients after bariatric surgery were less likely to drink soft drinks regularly (*p* = 0.026). Obese patients, before or after bariatric surgery, are at risk for erosive dental wear. However, with sufficient education prior to surgery and consistent intake of vitamin and mineral supplements, significant erosive dental wear after bariatric surgery could be avoided. Regular dental examination should be included in the check-up and follow-up program.

## 1. Introduction

Among patients with poorly managed obesity, metabolic/bariatric surgery has been proven to be the most effective and durable therapy for obesity [1]. Obesity and bariatric surgery have been shown to be associated with a higher risk for dental wear [2,3], which is multifactorial: frequent consumption of soft drinks is associated with obesity and dental problems [4,5,6]; unhealthy food choices might have led to micronutrient deficiency before surgery, e.g., iron, vitamin D and calcium [7,8,9,10]; aversion to special foods and taste changes were often reported after surgery [11,12,13,14]; and the reduced intestinal absorptive surface area affected hormonal mediators, which can lead to micronutrient deficiency postoperatively, including lower vitamin D and calcium in serum [15,16,17].

Erosive tooth wear is defined as a chemical–mechanical condition with an increasing prevalence worldwide, which results in a loss of hard dental tissue [18,19]. The erosion of enamel, the outer surface layer of the teeth, leads to an exposure of the dentin [20]. Unprotected dentin creates hypersensitivity to physical stimuli, such as heat and cold [21]. With progressive degradation processes, exposure of the pulp and thus avitalization of the teeth can occur [20]. As an important biological factor, saliva buffers the pH in the oral cavity and decelerates the process of dental erosion [22]. Low pH is a risk factor for hypersensitivity and erosion: dietary acids (e.g., soft drinks) have been associated with erosive tooth wear [23,24], and a clear impact on erosion prevalence was found in patients with gastro-esophageal reflux (GERD) and eating disorders associated with vomiting [25]. In contrast, normal calcium concentration is considered as a major protective factor in determining the erosive potential [19].

GERD and vomiting are potential obesity-related problems and complications after bariatric surgery [26]. Considering the fact that patients are at further risk for calcium and vitamin D deficiency after bariatric surgery, it is suggested that the oral impactions of bariatric surgery might have consequences such as dental erosion. A recent review based on five Brazilian studies concluded that patients undergoing bariatric surgery had a higher incidence of dental complications [27]. The changes of saliva after bariatric surgery are also contradictory in the literature. Robust evidence, especially for dental erosion, is still lacking.

This study aimed to compare the dental erosion in obese patients before and after bariatric surgery. Potential dietary and health factors associating with erosive tooth wear were also evaluated.

## 2. Materials and Methods

### 2.1. Study Design

The study was designed as a single-center, cross-sectional analysis observing the effects of bariatric procedures on the severity of tooth erosion. The study was approved by the university faculty ethics committee and institutional review board (#2020-598N) and was conducted at a university hospital. The trial is registered in the German Clinical Trials Register (DRKS00025580).

### 2.2. Sample Size Calculation and Inclusion

Based on the described changes of dental wear and salivary flow after bariatric surgery in a previous study [28], we used G * Power sample size calculator [29] and set alpha to 0.05 and power to 0.9, resulting in a sample size of 30 participants per group.

The control group was composed of patients who possessed poorly managed obesity (body mass index (BMI) ≥ 35 kg/m^2^ and one or more comorbidities (e.g., diabetes, arterial hypertension, or sleep apnea), or BMI ≥ 40 kg/m^2^) who presented for bariatric surgery in the outpatient clinic; the surgery group consisted of patients who had already underwent bariatric surgery at least 3 months previously. Participation for all patients was predicated on their written informed consent.

### 2.3. Dental Examination

#### 2.3.1. BEWE Score

The BEWE (basic erosive wear examination) is a simple tool designed to assess the level of dental erosion [30]. The teeth are divided into sextants. Only the value of the tooth surface with the highest BEWE value per sextant is documented. A value from 0 to 3 is determined in each sextant. Table 1 shows the criteria for sextant scores from 0 to 3, which are summed to obtain a cumulative score (0–18), which is the basis for determining interventions. The BEWE categories define the severity of erosion in 4 groups: group 0: 0–2 points; group 1: 3–8 points; group 2: 9–13 points; and group 3: 14–18 points. The BEWE categories can be further divided into the low-risk group (group 0 and 1) and the high-risk group (group 2 and 3). Patients in the high-risk group require dental health interventions.

#### 2.3.2. Sialometry

To determine the saliva flow rate, a measurement of the unstimulated saliva production within 5 min as the “spitting method” was used [31]. Using the unstimulated method, the naturally produced saliva is gathered in the floor of the mouth and spat into a collecting tube at certain time intervals. A hyposalivation is defined as salivary flow below 0.25 mL/min.

Blood samples were collected on the same day of dental examination. Vitamins and minerals, including vitamin D and calcium in serum, were measured in a routine diagnostic setting. 

### 2.4. Questionnaire

Using an investigative questionnaire, the following data were collected: sociodemographic data (age, gender, education level, income, and migrant status), preexisting comorbidities (e.g., diabetes, arterial hypertension, and thyroid disease), dietary habits (soft drinks, eating frequencies, smoking, alcohol consumption and eating disorders), gastrointestinal discomforting (regurgitation, gastroesophageal reflux and vomiting) and dental health (hypersensitivity, history of dental or periodontal disease) as well as dental health awareness (dentist visit frequency and oral hygiene).

### 2.5. Statistical Analysis

All statistical calculations were performed with the RStudio Version 1.2.5042, “Double Marigold” (Boston, MA, USA) and Python 3.9.5. (Wilmington, DE, USA) For quantitative variables, the mean and standard deviations were assessed. For qualitative factors, absolute and relative frequencies were given. For non-normally distributed data, the Wilcoxon rank-sum test was used. Pearson correlation coefficient measured correlations between two metric variables, Spearman correlation between an ordinal and a metric variable, and Chi^2^-test between a nominal and an ordinal variable. In general, the result of a statistical test was considered statistically significant for a *p*-value < 0.05.

## 3. Results

The study was conducted at our university hospital between January and March 2021. All operations were performed laparoscopically by two of the co-authors (M.O. and G.V.) and the dental examinations were carried out by a dentist (co-author F.J.H.).

### 3.1. Demographics

Obese patients who presented for bariatric surgery or a follow-up after bariatric surgery were included. Sixty-two patients were enrolled (thirty-one in each group), with an average age of 40 years old. The majority of the participants are females. Participants in the control group are younger and more obese. Other sociodemographic characteristics did not differ between groups (Table 2). 

### 3.2. Oral Health Related Parameters

Patients after bariatric surgery were less likely to drink soft drinks regularly (*p* = 0.026) and reported less GERD (*p* = 0.012). Other parameters, including hypersensitivity of the teeth, eating disorders and vomiting, were similar among the groups (Table 3). 

### 3.3. Dental Examination and Level of Serum Vitamin D/Calcium

All patients after bariatric surgery have taken the recommended supplements, including vitamin D and calcium citrate, daily. Among all patients, three patients had a hypocalcaemia (<2.18 mmol/L), who were all in the control group. No hypocalcaemia was found in the surgery group. Vitamin D deficiency (<20 µg/L) was detected in 19 patients in the control group and three in the surgery group (*p* < 0.001). The serum calcium and vitamin D values were significantly higher in the surgery group (*p* = 0.003, *p* < 0.001 consecutively).

**Figure 1:**
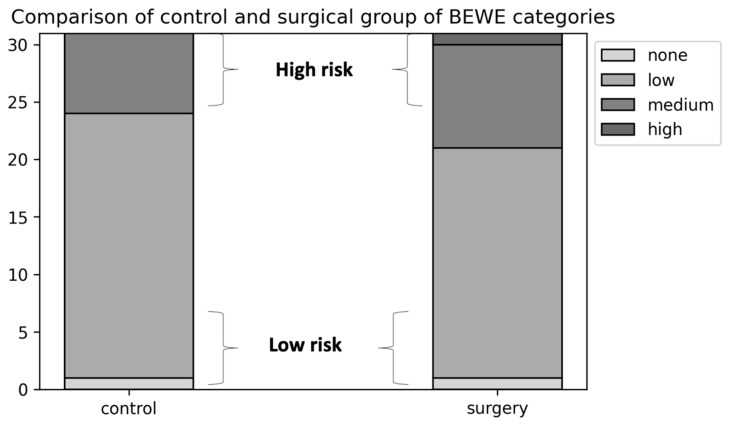
Portion of BEWE categories in control and surgery group.

BEWE scores, BEWE category and BEWE risk groups did not vary between groups. Of the preoperative obese patients, 22.6% were classified in BEWE category 2 and 3, therefore they had a high risk for further exposure of the pulp and consequential avitalization of the teeth (Figure 1). In the surgery group, the percentage was even higher (32.2%), but the difference was not significant. Neither was there a significant difference in the salivary flow (Table 4). 

### 3.4. Correlation

Sociodemographic parameters, such as age, education level, migration status and comorbidities, did not show significant correlations with BEWE scores, risk and salivary flow.

### 3.5. VSG versus RYGB 

In the surgery group, seven patients underwent a vertical sleeve gastrectomy (VSG), 21 underwent a Roux-en-Y gastric bypass (RYGB) and three others underwent a single-anastomosis duodeno-ileal bypass (S.A.D.I-S) or bilio-pancreatic diversion with duodenal switch (BPD-DS). The frequency of GERD, vomiting, BEWE scores, BEWE categories, risk, salivary flow, salivary flow categories, calcium and vitamin D did not differ between patients who underwent VSG and RYGB.

### 3.6. Short-Term Follow-Up versus Long-Term Follow-Up

In the surgery group, the period between bariatric surgery and dental examination was 11 (3–142) months. Patients with a shorter follow-up (<11 months) had significantly higher calcium levels in the serum (*p* = 0.014). Vitamin D, BEWE scores and salivary flow did not differ between patients with a short-term (<11 months) and long-term (>11 months) follow-up.

## 4. Discussion

The prevalence of obesity is increasing, along with the number of bariatric surgeries. Accordingly, the side effects of obesity and bariatric surgery are gaining growing attention. Several factors associated with bariatric surgery might lead to dental health problems: micronutrient deficiency, as vitamin D and calcium deficiency accompanies the great severity of oral disease [35,36]; increased prevalence of gastroesophageal reflux and vomiting, which lowers the pH in oral cavity, and is consequently a major risk factor for erosive dental wear [23,24]; and the postoperative, recommended, small yet frequent meals (4–6 meals/day), which shorten the regeneration period for the saliva [28,37], which is of great importance for the hard tissue protection. 

The current study confirmed that a significant number of obese patients are at a high risk for erosive dental wear and can experience further exposure of the pulp and thus avitalization of the teeth. However, the condition of dental wear did not worsen significantly after bariatric surgery. This is not in line with the limited data in the literature which have evaluated the effect of bariatric surgery on dental erosion. Quintella et al. reviewed five Brazilian studies and concluded that patients undergoing bariatric surgery had a higher incidence of dental wear [27]. Of these studies, one focused on erosive damage, and showed more severe dental erosion in patients after bariatric surgery [38]. The divergence of conclusions might be multifactorial. Firstly, the entire population in our surgery group have taken recommended supplements, including calcium citrate and vitamin D3, which was confirmed by the significantly higher serum levels of calcium and vitamin D after surgery. Calcium and vitamin D are known to have a protective effect on hard tooth tissues [39]. The recommended supplement of vitamins and minerals was not mentioned in the Brazilian studies. Secondly, GERD and vomiting were not increased after bariatric surgery in our observation. On the contrary, GERD was significantly less reported in the surgery group, which can be explained by two facts: postoperative patients were less obese (significant lower BMI) and therefore possessed a decreased risk for GERD [40], and, in the majority of the postoperative patients, RYGB was performed. After RYGB, gastric reflux remission is more frequently observed than after VSG [41]. Thus, RGYB could be a protective factor for dental health. Thirdly, the daily consumption of soft drinks, another potential factor to lower pH in oral cavity, was significantly reduced in our surgery group. Previous investigations showed that soft drink consumption can contribute to detrimental oral health, especially due to the erosive potential [6]. Postoperative taste changes might explain the altered dietary habits [11,13]. Moreover, patients were required to take part in a minimally 6-month long, multimodal concept, including intensive consultation by nutritional therapist before the surgery. Nutritional therapists assess and correct their nutritional status and, more importantly, educate the patients on how to establish healthy dietary habits (fewer soft drinks, less carbohydrates, and more proteins, etc.). The presurgical education seemed to have a lasting effect in the patients, which was not mentioned in the Brazilian studies. Fourthly, the spans between the bariatric surgery and the survey differ. Interestingly, studies confirming a worsening of dental wear in patients after bariatric surgery were performed with a relatively short flow-up (3–6 months) [28,42]. Even with a longer follow-up span (with a median of 11 months), no significant difference was detected in our study population. Moreover, patients with a longer follow-up did not present with a higher risk for erosive dental wear than patients with a short-term follow-up in our study. It can be assumed that the risk of erosive dental wear would not increase if patients were compliant with supplements and follow-up.

Saliva is an important biological factor, which buffers the pH in the oral cavity and decelerates the process of dental erosion [22]. The impact of bariatric surgery on saliva production remains unclear. Some studies showed an improvement in the salivary flow rate, while others found no change or even a worsening in saliva production after the surgery [43]. In our study, the salivary rate did not differ between the groups. The inconsistency might be partially due to different measuring methods. We preferred the unstimulated spitting method to the stimulated measurement, since the unstimulated saliva flow, which occurs around 14 to 16 h per day, is primarily responsible for the maintenance of oral health and the protection of our teeth. The stimulated saliva flow rate, on the other hand, embodies the functional capacity of the salivary glands and is only present for around two hours a day [31]. Furthermore, our measurement was performed in two different groups, and interindividual differences might preexist for salivary production.

To the best of our knowledge, this is the first study evaluating erosive dental ware in obese patients before and after bariatric surgery in Europe. Bariatric surgery might be associated with risks of erosive dental wear due to multiple factors. However, with sufficient education prior to surgery, consistent intake of vitamin and mineral supplements, and regular follow-ups, significant erosive dental wear after bariatric surgery could be avoided. Regular dental examination should be included in the follow-up program after bariatric surgery. A remineralization solution might help to prevent dental erosions from occurring [44].

## 5. Limitations

The majority of patients in the surgery group received a Roux-en-Y gastric bypass. The results might be different in patients after sleeve gastrectomy. Overall, there are more female than male participants in our study. Patients were not perfectly paired in both groups due to different BMIs and ages. To minimize the bias, dental examination should be performed in patients indicated for bariatric surgery before and after surgery in further trials. 

## 6. Conclusions

Obesity and bariatric surgery might be associated with risks for erosive dental wear due to multiple factors. However, with sufficient education prior to surgery and consistent intake of vitamin and mineral supplements, significant erosive dental wear after bariatric surgery could be avoided. Regular dental examination should be included in the check-up and follow-up program in obese patients before and after bariatric surgery.

## Figures and Tables

**Table 1:** BEWE (basic erosive wear examination) scores and criteria.

Score	Criteria
0	No erosion
1	Initial loss of surface texture
2	Distinct defect; hard tissue loss involving <50% of the surface area
3	Hard tissue loss ≥50% of the surface area

**Table 2:** Sociodemographic characteristics of 62 patients.

Variables	Control	Surgery	*p*-Value
Number of patients	31	31	n.s.
Female (%)	22 (71)	28 (90)	n.s.
Age (years), Mean ± SD	37.5 ± 9.6	42.7 ± 10.1	*p* = 0.038
BMI (kg/m^2^), Mean ± SD	46.1 ± 7.5	32.5 ± 5.9	*p* < 0.0001
Education classification (%)			n.s.
low	11 (35.5)	7 (22.4)	
middle	16 (51.6)	16 (51.6)	
high	4 (12.9)	8 (25.8)	
Income (%)			n.s.
low	6 (19.4)	3(9.7)	
middle	22(71.0)	20 (64.5)	
high	3(9.6)	8 (25.8)	
Migration (%)	8 (25.8)	3 (9.7)	n.s.

BMI, body mass index; n.a., not available; n.s., not significant; SD, Standard Deviation. Significant *p*-values are highlighted in bold (Wilcoxon, Chi^2^ and Fisher’s exact tests, *p* < 0.05). The education was classified based on ISCED 2011 (International Standard Classification of Education [32]). The income was classified by statistic data in Germany [33,34].

**Table 3:** Oral health related parameters.

Variables	Control	Surgery	*p*-Value
Smoking (%)	10 (32.2)	8 (25.8)	n.s.
Alcohol consumption (%)	2 (6.4)	1 (3.2)	n.s.
Soft drinks (%)	14 (45.2)	5 (16.1)	*p* = 0.026
Eating frequency			n.s.
1–3 meals/d (%)	22 (71.0)	15 (48.4)	
≥4 meals/d (%)	9 (29.0)	16 (51.6)	
Eating disorder (%)	6 (19.4)	4 (12.9)	n.s.
GERD (%)			*p* = 0.012
never	15 (48.4)	26 (83.9)	
maximal once/week	11 (35.5)	3 (9.7)	
>once/week	5 (16.1)	2 (6.4)	
Vomiting (%)			n.s.
never	28 (90.3)	27 (87.1)	
maximal once/week	3 (9.7)	3 (9.7)	
>once/week	0 (0)	1 (3.2)	
Hypersensitivity of teeth (%)	12 (38.7)	11 (35.5)	n.s.

GERD, gastroesophageal reflux disease; n.s., not significant. Significant *p*-values are highlighted in bold (Chi^2^ and Fisher’s exact tests).

**Table 4:** Results of dental examinations.

Variables	Control	Surgery	*p*-Value
Calcium, mmol/L	2.34 ± 0.11	2.42 ± 0.10	*p* = 0.003
Vitamin D, µg/L	18.0 ± 9.2	31.0 ± 12.1	*p* < 0.001
BEWE score	7.0 ± 2.2	7.4 ± 2.8	n.s.
BEWE category (%)			n.s.
0	1 (3.2)	1 (3.2)	
1	23 (74.2)	20 (64.5)	
2	7 (22.6)	9 (29.0)	
3	0 (0)	1 (3.2)	
BEWE risk categories (%)			n.s.
low risk	24 (77.4)	21 (67.7)	
high risk	7 (22.6)	10 (32.2)	
Salivary flow, mL/min	0.32 ± 0.1	0.32 ± 0.1	n.s.
Classification of salivary flow			n.s.
hyposalivation (%)	10 (32.2)	10 (32.2)	
normal salivary flow (%)	21 (67.8)	21 (67.8)	

BEWE (basic erosive wear examination); n.s., not significant. All values are shown as means and standard deviation or frequency and percentage. Significant *p*-values (*p* < 0.05) are highlighted in bold.

## Data Availability

The data presented in this study are available on request from the corresponding author.
